# The C_2_H_2_ Transcription Factor Con7 Regulates Vegetative Growth, Cell Wall Integrity, Oxidative Stress, Asexual Sporulation, Appressorium and Hyphopodium Formation, and Pathogenicity in *Colletotrichum graminicola* and *Colletotrichum siamense*

**DOI:** 10.3390/jof10070495

**Published:** 2024-07-17

**Authors:** Shuangzhen Zhou, Shayu Liu, Chenchen Guo, Hanwen Wei, Zhihui He, Zhiqiang Liu, Xiaoyu Li

**Affiliations:** School of Life and Health Sciences, Hainan University, Haikou 570228, China

**Keywords:** *Colletotrichum graminicola*, *Colletotrichum siamense*, C_2_H_2_ transcription factors, conidiation, appressorium formation

## Abstract

The *Colletotrichum* genus is listed as one of the top 10 important plant pathogens, causing significant economic losses worldwide. The C_2_H_2_ zinc finger protein serves as a crucial transcription factor regulating growth and development in fungi. In this study, we identified two C_2_H_2_ transcription factors, CgrCon7 and CsCon7, in *Colletotrichum graminicola* and *Colletotrichum siamense*, as the orthologs of Con7p in *Magnaporthe oryzae*. Both CgrCon7 and CsCon7 have a typical C_2_H_2_ zinc finger domain and exhibit visible nuclear localization. Disrupting *Cgrcon7* or *Cscon7* led to a decreased growth rate, changes in cell wall integrity, and low tolerance to H_2_O_2_. Moreover, the deletion of *Cgrcon7* or *Cscon7* dramatically decreased conidial production, and their knockout mutants also lost the ability to produce appressoria and hyphopodia. Pathogenicity assays displayed that deleting *Cgrcon7* or *Cscon7* resulted in a complete loss of virulence. Transcriptome analysis showed that CgrCon7 and CsCon7 were involved in regulating many genes related to ROS detoxification, chitin synthesis, and cell wall degradation, etc. In conclusion, CgrCon7 and CsCon7 act as master transcription factors coordinating vegetative growth, oxidative stress response, cell wall integrity, asexual sporulation, appressorium formation, and pathogenicity in *C. graminicola* and *C. siamense*.

## 1. Introduction

The *Colletotrichum* genus, comprising 200 members, is listed as one of the top 10 important plant pathogens, and can infect more than 3000 species of monocotyledons and dicotyledons, causing great economic losses all over the world [1,2]. *Colletotrichum graminicola* is the pathogen causing anthracnose leaf blight and stalk rot in maize, which has led to serious damage to the maize industry in recent years [3]. *C. graminicola* usually produces two kinds of conidia, namely, falcate conidia and oval conidia [4]. Oval conidia are generally formed inside the host and primarily related to intra-tissue spread [4]. Falcate conidia are primary infective spores that can individually produce germ tubes and then form appressoria to penetrate the host [5]. *C. siamense*, which is a member of the *C. gloeosporioides* species complex, is the main causal agent of rubber anthracnose [6]. *C. siamense* invades the epidermis of rubber tree leaves, posing a threat to the growth of rubber trees and natural rubber production [6]. Like the falcate conidium of *C. graminicola*, the conidium of *C. siamense* generates a germ tube and forms an appressorium at its top after adhering to leaves. Then, the appressorium produces a penetration peg and invades the epidermis of leaves [7]. In some *Colletotrichum* species, the hyphopodium is usually developed at mature hyphal tips, which also plays a role in the penetration of the plant epidermis [8]. Therefore, conidia serve as a primary dissemination tool and play an important role in the infection of the two pathogenic fungi [9,10]. In-depth analyses of the regulatory mechanisms of conidial production and infection have important theoretical and practical significance for understanding the interactions between pathogens and hosts and thus potentially for effectively controlling diseases.

For many pathogenic fungi, the colonization in hosts usually comprises conidial adhesion, germination, appressorium formation, and invasive growth, which is precisely regulated by transcription factors (TFs) [11]. The C_2_H_2_ zinc finger protein is a common TF, and plays a vital role in coordinating growth and development in fungi [12]. Con7-like TFs are typical C_2_H_2_ zinc finger proteins. Con7 was first characterized in *Magnaporthe oryzae*, where *con7* mutants produced abnormal conidia with significantly reduced conidiation [13]. Further research showed that Con7p was also required for appressorium formation in *M. oryzae* [14,15]. In *Verticillium* species, the Con7 ortholog Vta2 is a positive regulator of vegetative growth, conidiation, H_2_O_2_ detoxification, and virulence [16]. In *Fusarium oxysporum*, deleting *con7-1* resulted in non-pathogenic mutants, which also exhibited defects in hyphal branching, asexual sporulation, and cell wall structure [17]. In *F. graminearum*, disrupting *FgCON7* also led to severe defects in growth, asexual/sexual development, and virulence [18]. Overall, the Con7-like TFs act as central regulators in the processes of growth, development, stress response, and infection.

In this study, two Con7-ortholog TFs, CgrCon7 in *C. graminicola* and CsCon7 in *C. siamense*, were functionally characterized through genetic manipulations. We found that both CgrCon7 and CsCon7 were implicated in regulating vegetative growth, oxidative stress response, cell wall integrity, conidiation, appressorium formation, and virulence. Furthermore, the regulatory networks of the two TFs were also elucidated by transcriptome analysis.

## 2. Materials and Methods

### 2.1. Fungal Strains and Culture Conditions

In this study, *C*. *graminicola* strain CgM2 (CgrWT) and *C*. *siamense* strain CsWT were used as wild-type strains and preserved in the School of Life and Health Sciences, Hainan University. The wild-type strains and their derivative strains were routinely maintained on potato dextrose agar (PDA) for 5 days at 28 °C and then stored at 4 °C. The media used in this work, such as PDA, CM, MM, CZAPEK (CZ), LB, DCM, and TB3, were prepared as described previously [19]. To evaluate vegetative growth, all strains were inoculated on PDA, CM, CZ, and MM media at 28 °C, and colony diameters were measured 5 days post-incubation (dpi).

### 2.2. Nucleic Acid Manipulations and Sequence Analysis

The genomic DNA (gDNA) of *C. graminicola* and *C. siamense* was obtained using the CTAB method [20]. Total RNA extraction and cDNA synthesis were performed as previously described [21]. The primer pairs Cgrcon7F–Cgrcon7R and Cscon7F–Cscon7R were used for amplifying the open reading frames (ORFs) of *Cgrcon7* and *Cscon7* from the cDNAs, respectively. The protein domain was analyzed on the SMART platform (http://smart.embl-heidelberg.de/, accessed on 18 March 2024). The BLAST tool (https://blast.ncbi.nlm.nih.gov/Blast.cgi, accessed on 18 March 2024) in GenBank was used to search Con7 homologous protein sequences in different fungi. Protein sequence alignment and motif analysis were carried out as previously described [22].

### 2.3. Target Disruption and Complementation

Cg*rcon7* and *Cscon7* were disrupted using the homologous recombination method (Figure S1). For deleting *Cgrcon7*, the primer pairs Cgrcon7UF–Cgrcon7UR and Cgrcon7DF–Cgrcon7DR were used to amplify 1108 bp-upstream and 1003 bp-downstream flanking fragments from the gDNA of *C. graminicola*, respectively. The two fragments were ligated to a vector pUC18–HPT (pUC18 harboring the hygromycin phosphotransferase gene *HPT*). Then, the linearized plasmid was transformed into CgrWT protoplasts and the transformation was performed as described previously [23]. Transformants were screened and verified by PCR using the primer pairs Cgrcon7F–Cgrcon7R, Cgrcon7UU–pUC18–PI and pUC18–PI1–Cgrcon7DD (Figure S1A). The primers Cgrcon7hbF and Cgrcon7hbR were used to amplify the complementary fragment of *Cgrcon7*, which was inserted into the pUC18 vector fused with a neomycin resistance gene. The complementary vector was transformed into protoplasts derived from a *Cgrcon7*-deletion mutant. Then, transformants were selected on a medium containing G418, and the primers Cgrcon7F and Cgrcon7R were used for PCR validation.

The gene deletion and complementation of *Cscon7* were performed as previously described [21]. Briefly, the 5′ and 3′ flank sequences of *Cscon7* were inserted into pCB1532 and then transformed into protoplasts from *C. siamense* CsWT. Transformants were screened and validated using Cscon7F–Cscon7R, Cscon7UU–PI, and PI1–Cscon7DD (Figure S1B). The complementary fragment of *Cscon7* was inserted into the pUC18–HPT vector and then transformed into protoplasts prepared from a *Cscon7*-deletion mutant. Then, transformants were screened on the TB3 agar medium containing hygromycin B, and Cscon7F and Cscon7R were used for PCR verification. The primers used in this section are listed in Table S1.

### 2.4. Subcellular Localization

To analyze the subcellular localization of CgrCon7, the *trpC* promoter, *Cgrcon7*-coding sequence, *eGFP*-coding sequence, and *trpC* terminator were fused and inserted into the vector pUC18–HPT. The recombinant plasmid was transformed into protoplasts from strain CgrWT. For *C*. *siamense*, the construction of the *Cscon7*–*eGFP* fusion fragment was similar to that of *Cgrcon7*. The fragment was ligated to pCB1532 and transformed into protoplasts from strain CsWT. The transformation was performed exactly as described previously [24]. The mycelia and conidia from transformants were observed using a fluorescent microscope (Leica DM LB2, Leica Microsystems Inc., Wetzlar, Germany). Nuclei were visualized using 4′,6-diamidino-2-phenylindole (DAPI) (10 μg/mL) (Sigma-Aldrich, St. Louis, MO, USA).

### 2.5. Stress Response Assays

To test the cell wall integrity (CWI), mycelial plugs from the wild type, gene-deletion mutants, and complementary strains were inoculated on MM plates with SDS and congo red (CR). The colony diameter was determined on 7 dpi, and inhibition rates were calculated as described previously [19]. For calcium fluorescent white (CFW) staining, the mycelial plugs from different strains were placed on glass slides and incubated at 28 °C. After 3 days, hyphae were stained with CFW staining solution containing 1 g/L CFW and 1 M NaOH (*v*/*v* = 1:1) for 5 min in the dark and visualized using fluorescence microscopy [22].

For H_2_O_2_-sensitive assays, strains were incubated in the PDB medium at 28 °C and conidia were obtained by filtration on 3 dpi. Fifteen milliliters of PDA medium was mixed with 100 μL conidial suspension (1 × 10^6^ conidia/mL) to pour a plate. Then, a filter paper disk soaked with 30% H_2_O_2_ was placed in the center of the plate. The plated was cultured at 28 °C, and the diameter of the inhibition zone was determined [16].

### 2.6. Conidiation, Germination, and Appressorium Formation

To obtain falcate conidia of *C. graminicola*, the strains were cultured on oatmeal agar (OMA: 50 g/L oatmeal, 18 g/L agar) for 14 days at 28 °C. The falcate conidia were rinsed using 5 mL of sterile water, and the conidial yield was determined using a hemocytometer. Regarding oval conidia, the strains were cultured in PDB for 48 h at 28 °C. Miracloth (Calbiochem, San Diego, CA, USA) was used for removing hypha of the sampled culture, and conidial yields were counted using the hemocytometer. The collection and determination of conidia from *C. siamense* were similar to those of oval conidia from *C. graminicola*.

For conidial germination, 20 μL of conidial suspension (5 × 10^5^ conidia/mL) was dropped on cellophane and kept at 28 °C. Then, conidial germination and appressorium formation were observed under a microscope. The percentages of germination or appressorium formation were determined at desired time points. To observe the formation of hyphopodia, mycelial plugs were inoculated onto glass slides and cultured at 28 °C, and the hyphopodium was observed using a microscope on 3 dpi.

### 2.7. Virulence Assay

The inoculation assay of *C. graminicola* strains was performed using susceptible maize (*Zea mays* L.) cultivar Xianyu 335. Three-week-old maize leaves were selected and wounded using a sterile needle. Mycelial plugs (5 mm diameter) or 20 μL of falcate conidial suspensions (5 × 10^5^ conidia/mL) of the strains were inoculated onto maize leaves, and mock inoculation was performed with agar plugs or sterile water, respectively. The virulence assay of *C. siamense* was similar to that of *C. graminicola*, except for using detached rubber tree leaves as inoculation objects. The leaves were kept in a moist chamber (relative humidity > 90%), and the severity of disease symptoms was recorded on 3 dpi.

### 2.8. RNA Sequencing

Transcriptome analysis of Δ*Cgrcon7* (Δ*Cscon7*) and CgrWT (CsWT) was performed by RNA sequencing (RNA-Seq). For *C. graminicola*, the wild type and Δ*Cgrcon7* were cultured on PDA medium at 28 °C for 5 days, and their mycelia were collected to extract total RNAs. Both the CgrWT and Δ*Cgrcon7* samples had three repeats. The RNA-Seq was performed by BGI (Wuhan, China) using a MGISEQ-2000 platform. The genome of *C. graminicola* M1.001 from GenBank was used as a reference. Data processing and analysis were based on a previous method [21]. The RNA-Seq of *Cscon7* was similar to that of *Cgrcon7*, and the genome of *C. siamense* Cg363 from GenBank was used as a reference. The RNA-Seq data were verified using quantitative RT-PCR (qRT-PCR) [25].

### 2.9. Statistical Analysis

All experiments were performed in triplicate, and the results are expressed as the mean ± standard deviation. SPSS software 20.0 was used for data analyses. One-way ANOVA test and Duncan’s multiple comparisons were used for significant difference analysis (*p* < 0.05 and *p* < 0.01), and GraphPad Prism 8.0 was used for graph making.

## 3. Results

### 3.1. Characterization of CgrCon7 and CsCon7

**Figure 1:**
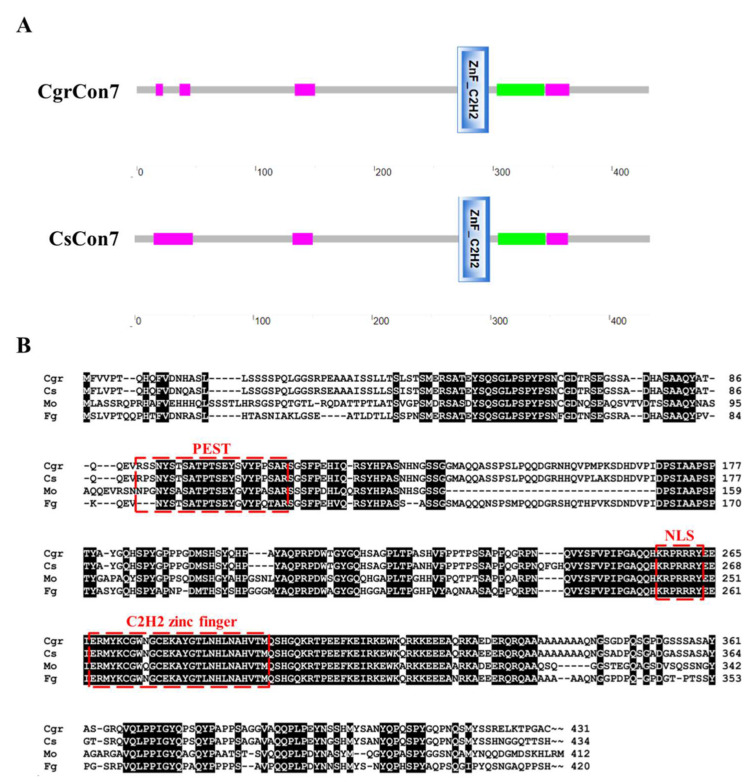
Bioinformatic analyses of CgrCon7 and CsCon7. (**A**) Protein domain analyses of CgrCon7 and CsCon7. The magenta box represents a low-complexity region. The green box represents a coiled-coil region. (**B**) Comparative alignment between deduced *Colletotrichum* Con7 protein sequences and orthologs from other plant pathogens. The deduced Con7 proteins were predicted to share a conserved C_2_H_2_ zinc finger domain, a nuclear localization signal (NLS), and a PEST motif for protein degradation. Cgr: *Colletotrichum graminicola* (XP_008089279.1), Cs: *Colletotrichum siamense* (XP_036502431.1), Mo: *Magnaporthe oryzae* (XP_003712849.1), Fg: *Fusarium graminearum* (XP_011321497.1).

A 1296-bp ORF of *Cgrcon7* was amplified from the cDNA of *C. graminicola* CgrWT, which encodes the 431-amino acid (aa) protein CgrCon7 (GenBank accession number: XP_008089279.1). The ORF of *Cscon7* is 1305 bp in length, encoding the 434-aa protein CsCon7 (XP_036502431.1). Multiple sequence alignments revealed that all Con7 orthologs harbored a highly conserved C_2_H_2_ zinc finger domain, as well as a nuclear localization signal (NLS), a PEST motif for protein degradation and a coiled-coil region responsible for protein–protein interactions (Figure 1A,B) [26,27]. CgrCon7 has high similarity with CsCon7, with an identity of 94.1%, and it also shares 72.7% and 63.5% identity with *F. graminearum* Con7 (XP_011321497.1) and *M. oryzae* Con7p (XP_003712849.1), respectively.

**Figure 2:**
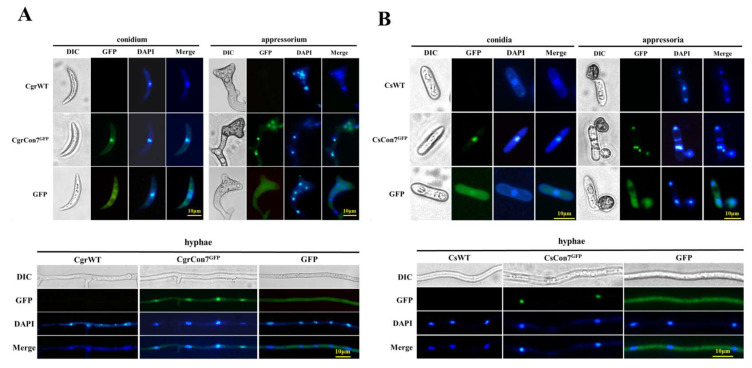
Subcellular localization of CgrCon7 and CsCon7 in the conidium, appressorium, and hyphae. (**A**) Subcellular localization of CgrCon7 in *C. graminicola*. (**B**) Subcellular localization of CsCon7 in *C. siamense*.

Fluorescence microscopy examination displayed that both CgrCon7 and CsCon7 were mainly localized in the nucleus of hyphae, conidia, and appressoria, with a small distribution in the cytoplasm (Figure 2A,B). The expression levels of *Cgrcon7* and *Cscon7* were investigated by qRT-PCR at different growth stages. With the increase in culture time, the expression levels of two genes increased continuously. Furthermore, both *Cgrcon7* and *Cscon7* exhibited high expression levels in conidia (Figure S2A,B).

### 3.2. Gene Disruption and Complementation of Cgrcon7 and Cscon7

To study the biological function of CgrCon7 and CsCon7, we generated *Cgrcon7*- and *Cscon7*-knockout mutants using the homologous replacement method. For the deletion of *Cgrcon7* in *C. graminicola*, the validation results displayed that transformants 10, 13, and 17 had a specific amplification band using Cgrcon7UU–pUC18–PI and pUC18–PI1–Cgrcon7DD (Figure S3B,C), whereas no target band was amplified using Cgrcon7F–Cgrcon7R (Figure S3A). The three transformants were named Δ*Cgrcon7*-10, Δ*Cgrcon7*-13 and Δ*Cgrcon7*-17, respectively. Regarding the disruption of *Cscon7* in *C. siamense*, two *Cscon7*-knockout mutants were obtained using the same method and named Δ*Cscon7*-4 and Δ*Cscon7*-11 (Figure S3D–F). For complementation, the gDNA sequences of *Cgrcon7* and *Cscon7* were retransformed into their corresponding disruption mutants, and their complementary strains were named Δ*Cgrcon7*-C and Δ*Cscon7*-C, respectively.

### 3.3. Con7 Is Required for Vegetative Growth in C. graminicola and C. siamense

**Figure 3:**
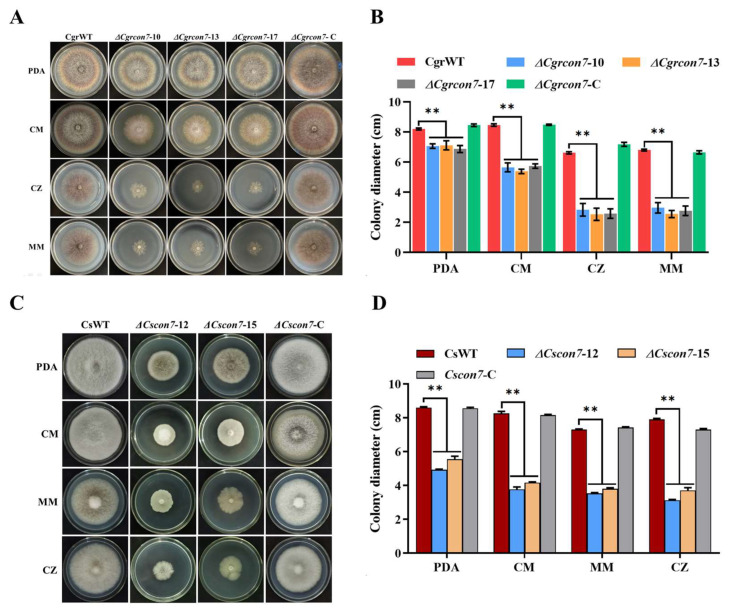
Vegetative growth on four media. (**A**) Growth comparison of *C. graminicola* strains on four media. (**B**) Statistical analyses of colony diameters of *C. graminicola* strains. (**C**) Growth comparison of *C. siamense* strains on four media. (**D**) Statistical analyses of colony diameters of *C. siamense* strains. ** Significant at *p* < 0.01.

The strains were cultured on PDA, CM, CZ, and MM media to evaluate vegetative growth. As shown in Figure 3, all deletion mutants of *Cgrcon7* and *Cscon7* decreased in growth rate significantly on the four media. For instance, the diameter of ΔCgrcon7 was about 2.6 cm on the CZ medium, whereas the diameter of CgrWT was up to 6.6 cm. The growth rates of Δ*Cgrcon7* and Δ*Cscon7* were less than 50% those of the wild-type strains on CZ and MM media. These results show that Con7 is involved in regulating the vegetative growth of *C. graminicola* and *C. siamense*.

### 3.4. Con7 Regulates CWI and Oxidative Stress in C. graminicola and C. siamense

**Figure 4:**
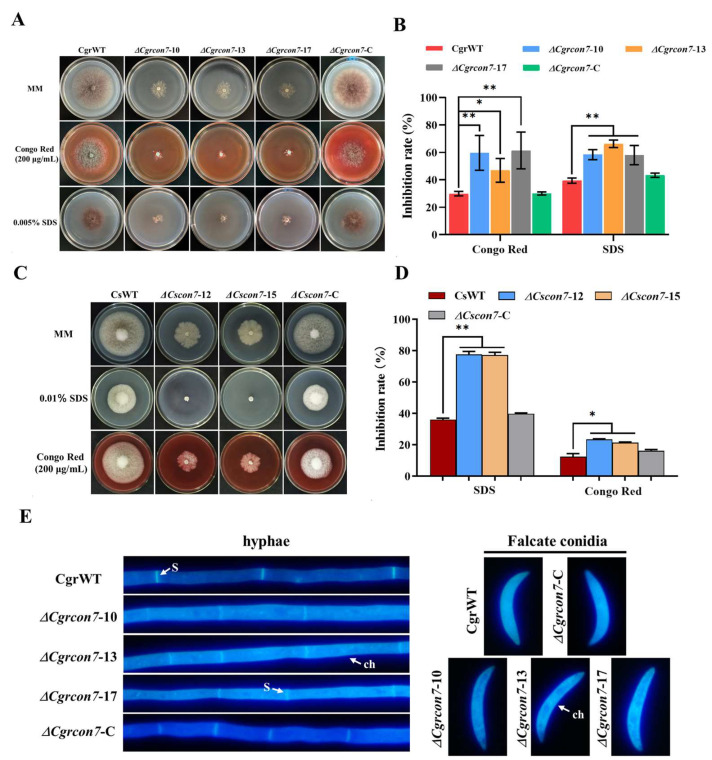
Effects of Con7 on the cell wall integrity of *C. graminicola* and *C. siamense*. (**A**) Effects of SDS and congo red (CR) on the growth of *C. graminicola* strains. (**B**) Statistical analyses of inhibition rates of *C. graminicola* strains. (**C**) Effects of SDS and CR on the growth of *C. siamense* strains. (**D**) Statistical analyses of inhibition rates of *C. siamense* strains. * Significant at *p* < 0.05; ** significant at *p* < 0.01. (**E**) CFW dyeing of the mycelia and falcate conidia of *C. graminicola* strains. s: septa; ch: chitin.

To investigate the role of CgrCon7 in CWI, all the strains were cultured on media supplemented with SDS and CR. The results showed that the *Cgrcon7*-deletion mutants were more sensitive to SDS and CR than CgrWT (Figure 4A,B). Δ*Cscon7* was similar to Δ*Cgrcon7*, exhibiting elevated sensitivity to two stress factors (Figure 4C,D). Furthermore, the distribution of chitin was investigated by CFW dyeing in *C. graminicola*. In the hyphae and conidia of CgrWT, chitin was located uniformly in the cell wall and concentrated in the septa of hyphae. In contrast, chitin displayed enhanced distribution in the cell wall of Δ*Cgrcon7* and also exhibited punctate distribution in some areas of hyphae and falcate conidia (Figure 4E). The above results suggest that CgrCon7 and CsCon7 participate in the regulation of cell wall integrity.

**Figure 5:**
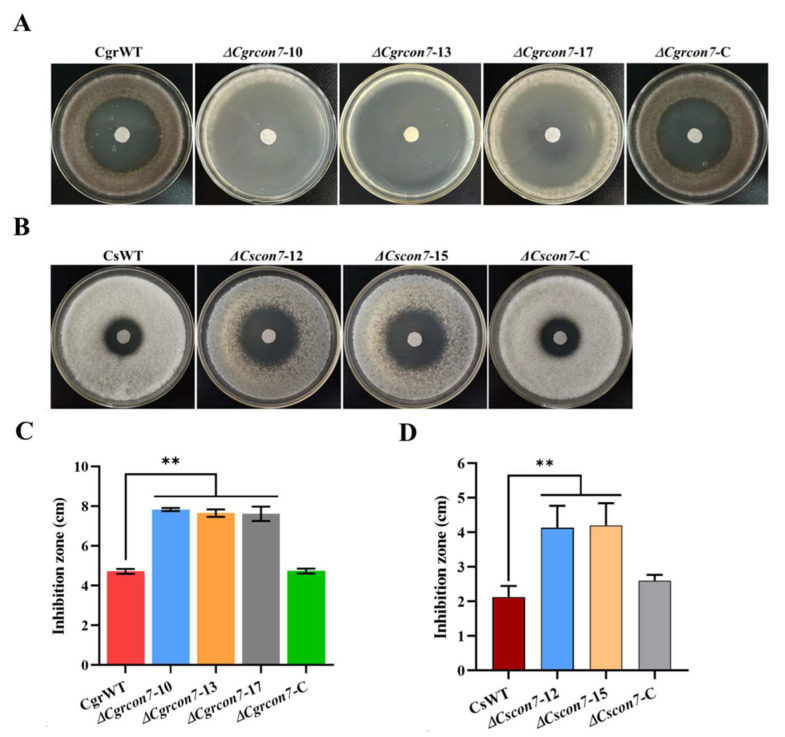
H_2_O_2_-sensitive assays. (**A**) Effects of H_2_O_2_ on the growth of *C. graminicola* strains on 3 dpi. (**B**) Effects of H_2_O_2_ on the growth of *C. siamense* strains on 2 dpi. (**C**) Statistical analyses of inhibition zone diameters of *C. graminicola* strains. (**D**) Statistical analyses of inhibition zone diameters of *C. siamense* strains. ** Significant at *p* < 0.01.

The H_2_O_2_-sensitivity assay was used to test whether Con7 affects oxidative stress in *C. graminicola* and *C. siamense*. As shown in Figure 5A,B, the filter paper had formed apparent inhibition zones on all the plates inoculated with different strains. The diameters of inhibition zones caused by Δ*Cgrcon7* and Δ*Cscon7* were significantly larger than those of the wild-type strains (Figure 5C,D). Deleting *Cgrcon7* or *Cscon7* resulted in apparent defects in H_2_O_2_ detoxification. Therefore, Con7 is involved in the regulation of the oxidative stress response in *C. graminicola* and *C. siamense*.

### 3.5. Con7 Is Involved in Conidiation, Conidial Morphology, Appressorium, and Hyphopodium Formation

**Figure 6:**
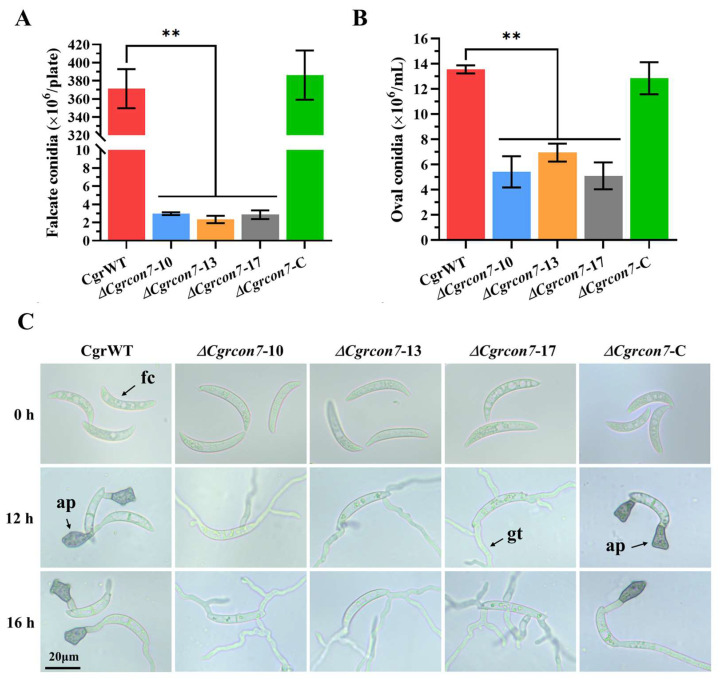
Conidial production and germination of *C. graminicola* strains. (**A**) Statistical analyses of the falcate conidium yield of *C. graminicola* strains. (**B**) Statistical analyses of the oval conidium yield of *C. graminicola* strains. ** Significant at *p* < 0.01. (**C**) Falcate conidium germination of *C. graminicola* strains. fc: falcate conidium, gt: germ tube, ap: appressorium.

For falcate conidia, the *C. graminicola* strains were cultured on OMA medium, and the conidial production was determined on 14 dpi. It was found that the yield of falcate conidia from Δ*Cgrcon7* was about 1% that of CgrWT (Figure 6A). We further determined the oval conidium yield of Δ*Cgrcon7*, which was also significantly lower than that of CgrWT (Figure 6B). Moreover, deleting *Cgrcon7* made the falcate conidium more slender, and the falcate conidium of Δ*Cgrcon7* became longer and thinner than that of CgrWT (Figure S4A,B). There was no dramatic difference in the germination of falcate conidia or oval conidia between Δ*Cgrcon7* and CgrWT. However, the falcate conidia from Δ*Cgrcon7* did not form appressoria normally on cellophane, whereas CgrWT had produced melanized appressoria at 12 h postinoculation (Figure 6C).

**Figure 7:**
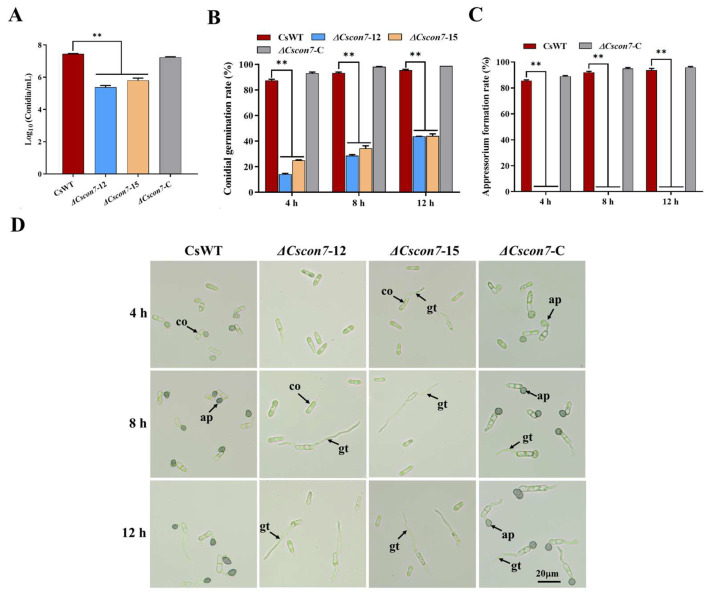
Conidial production and germination of *C. siamense* strains. (**A**) Statistical analyses of the conidium yield of *C. siamense* strains. (**B**) Statistical analyses of the conidial germination rate. (**C**) Statistical analyses of the appressorium formation rate of *C. siamense* strains. ** Significant at *p* < 0.01. (**D**) Conidial germination of *C. siamense* strains. co: conidium, gt: germ tube, ap: appressorium.

As for *C. siamense*, deleting *Cscon7* also led to distinctly decreased conidial production, and Δ*Cscon7* exhibited lower germination rates than CsWT (Figure 7A,B). Furthermore, Δ*Cscon7* did not produce appressoria, the same as Δ*Cgrcon7* (Figure 7C,D). The conidia of Δ*Cscon7* were smaller in length and width than those of CsWT (Figure S4C,D). We further detected the formation of hyphopodia in *C. graminicola* and *C. siamense*, and the results showed that the mycelia of Δ*Cgrcon7* and Δ*Cscon7* did not form hyphopodia normally on glass slides (Figure S5A,B). These findings suggest that Con7 is required for conidiation, maintaining conidial morphology, appressorium, and hyphopodium formation in *C. graminicola* and *C. siamense*.

### 3.6. Con7 Is Required for the Virulence of C. graminicola and C. siamense

**Figure 8:**
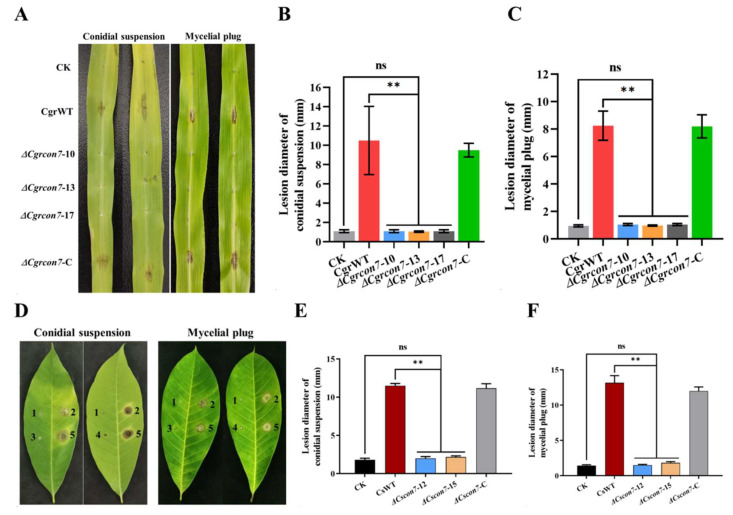
Virulence assays. (**A**) Disease symptoms on maize leaves using conidial suspension or mycelial plug inoculation of CgrWT, the Δ*Cgrcon7* mutants, and the complementary strain. Statistical analyses of lesion diameters for conidial suspension (**B**) and mycelial plug (**C**) inoculation of the *C. graminicola* strains. (**D**) Disease symptoms on rubber tree leaves using conidial suspension or mycelial plug inoculation of CsWT, the Δ*Cscon7* mutants, and the complementary strain. 1: CK, 2: CsWT, 3: Δ*Cscon7*-12*,* 4: Δ*Cscon7*-15, 5: Δ*Cscon7*-C. Statistical analyses of lesion diameters for conidial suspension (**E**) and mycelial plug (**F**) inoculation of the *C. siamense* strains. ** Significant at *p* < 0.01.

To determine the role of *Cgrcon7* in the virulence of *C. graminicola*, the CgrWT, Δ*Cgrcon7*, and complementary strains were inoculated on maize leaves. Regardless of the inoculation mode, typical and enlarged lesions were observed on the maize leaves inoculated with the wild type and complementary strain on 3 dpi. In contrast, there were limited lesions on the leaves inoculated with the Δ*Cgrcon7* mutants, and the lesion size showed no significant difference from that of the control group (Figure 8A–C). Subsequently, we determined the virulence of Δ*Cscon7* on rubber tree leaves. Compared with CsWT, Δ*Cscon7* did not form visible lesions on leaves and lost virulence completely (Figure 8D–F). These results indicate that Con7 is essential for the virulence of *C. graminicola* and *C. siamense*.

### 3.7. Transcriptomic Analysis of CgrCon7 and CsCon7

In order to analyze the regulatory network of CgrCon7 (CsCon7), the global RNA expression profiles of Δ*Cgrcon7* (Δ*Cscon7*) and CgrWT (CsWT) were compared. The selection for differentially expressed genes (DEGs) was established as a minimum of 2.0-fold down- or upregulation in the deletion mutant versus the wild type. For CgrCon7, 2907 genes were identified as DEGs, including 1588 upregulated genes and 1319 downregulated genes. Regarding CsCon7, 4124 genes displayed significant differences in expression levels, in which 1220 genes were upregulated and 2904 genes were downregulated. The RNA-Seq results of Δ*Cgrcon7* and Δ*Cscon7* were validated by qRT-PCR, and the expression levels of selected DEGs exhibited the same trend as those in RNA-Seq data, with all the correlation coefficients being greater than 96% (Figure S6).

**Figure 9:**
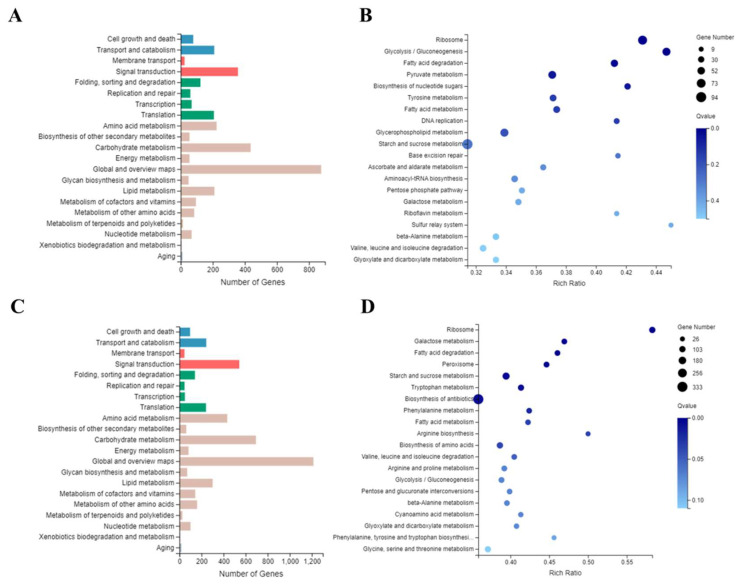
Functional classification and KEGG enrichment analyses of DEGs. (**A**) Functional classification of DEGs in the *Cgrcon7*-deletion mutant. (**B**) The top 20 enriched KEGG pathways of DEGs in the *Cgrcon7*-deletion mutant. (**C**) Functional classification of DEGs in the *Cscon7*-deletion mutant. (**D**) The top 20 enriched KEGG pathways of DEGs in the *Cscon7*-deletion mutant.

Then, we performed GO (Gene Ontology) function and KEGG (Kyoto Encyclopedia of Genes and Genomes) pathway analyses of DEGs from Δ*Cgrcon7* and Δ*Cscon7*. It was found that the functions of DEGs from two deletion mutants were mainly classified in (over 200 DEGs) transport and catabolism, signal transduction, translation, amino acid metabolism, carbohydrate metabolism, global and overview maps, and lipid metabolism (Figure 9A,C). The top 20 significantly enriched KEGG terms are listed in Figure 9B,D. The DEGs from Δ*Cgrcon7* and Δ*Cscon7* were both enriched in ribosome, fatty acid degradation, fatty acid metabolism, starch and sucrose metabolism, galactose metabolism, beta-alanine metabolism, valine, leucine, and isoleucine degradation, and glyoxylate and dicarboxylate metabolism. It was also noted that some enzyme genes related to ROS (reactive oxygen species) detoxification, chitin synthesis, and cell wall degradation were significantly affected due to the deletion of *Cgrcon7* or *Cscon7*, including superoxide dismutase, peroxidase, catalase, chitin synthase, cutinase, pectin lyase, glucanase, etc. (Table 1).

## 4. Discussion

The C_2_H_2_-type zinc finger is the most common DNA-binding motif in eukaryotes, especially in C_2_H_2_ TFs [28]. C_2_H_2_ TFs play an important role in regulating different signal transduction pathways and controlling various biological processes in eukaryotic cells. In this study, we identified and characterized two C_2_H_2_ zinc finger proteins, CgrCon7 and CsCon7, in *C. graminicola* and *C. siamense* that were orthologous to Con7p in *M. oryzae*. Con7-like TFs widely exist in filamentous fungi, but not in yeasts, suggesting that they may coordinate certain functions common to molds [15]. The spatiotemporal expression analysis showed that CgrCon7 and CsCon7 were mainly localized in the nucleus and expressed throughout the whole vegetative growth process, with high expression levels in conidia. The expression patterns suggest that CgrCon7 and CsCon7 may play crucial roles in the development of hyphae and conidia.

To investigate the functions of CgrCon7 and CsCon7, we constructed their deletion mutants in *C. graminicola* and *C. siamense*, respectively. Phenotypic analysis revealed that Δ*Cgrcon7* and Δ*cscon7* both exhibited notably decreased vegetative growth compared with the wild-type strains. The Con7 orthologs also displayed a positive regulatory role in the growth of *Verticillium dahliae, F. oxysporum*, and *F. graminearum* [16,17,29]. Based on the RNA-Seq results, we found that a large number of genes associated with the metabolism of carbohydrates, lipids, and amino acids were significantly influenced in Δ*Cgrcon7* and Δ*Cscon7*. Therefore, deleting *Cgrcon7* or *Cscon7* may cause metabolic disorders and further affect the vegetative growth of two *Colletotrichum* species. In *V. dahliae*, the RNA-Seq data showed that 34.5% of the downregulated genes in the *VTA2*-deletion mutant were involved in metabolism-related processes [16]. Moreover, numerous DEGs in the Δ*con7-1* mutant were also found to participate in regulating carbohydrate metabolism (133 genes), fatty acid metabolism (49), and amino acid metabolism (64) [17].

The fungal cell wall, which is typically composed of chitin and glucan, plays a crucial role in growth and development as well as adaptation to adverse environments [30,31]. In our study, deleting *Cgrcon7* (*Cscon7*) resulted in elevated sensitivity against SDS and CR, suggesting that Con7 is involved in maintaining CWI in *C. graminicola* and *C. siamense*. The KEGG analysis showed that the expression of over 300 genes in the mitogen-activated protein kinase (MAPK) signaling pathway were dramatically affected in Δ*Cgrcon7* or Δ*Cscon7*, and many of them were related to the CWI pathway. Furthermore, the CFW dyeing results showed that there were obvious differences in chitin distribution between Δ*Cgrcon7* and CgrWT, suggesting CgrCon7 may be involved in regulating chitin synthesis. The RNA-Seq data showed that the expression of two chitin synthase genes (GLRG_05787 and GLRG_03399) was affected to varying degrees by CgrCon7, and three chitin synthase genes were upregulated in the *Cscon7*-deletion mutant. Con7-like TFs have been reported to be connected with chitin metabolism. In *M. oryzae*, Con7p was involved in regulating the expression of genes related to cell wall biogenesis and remodeling, including the class VI chitin synthase Chs7 and the chitin-binding proteins Cbp1 and Cbp2 [32]. Furthermore, the chitin synthase gene (FGSG_06550) was significantly upregulated in the *FgCON7*-deletion mutant and affected the cell wall integrity of *F. graminearum* [18]. Overall, we speculate that Con7 participates in the regulation of chitin synthesis and further affects CWI in *C. graminicola* and *C. siamense*.

Conidia are important tools for the dissemination and infection of *C. graminicola* and *C. siamense*, and Con7 plays a crucial role in conidial production in the two pathogenic fungi. Disrupting *Cgrcon7* led to dramatically reduced conidiation, and the yield of falcate conidia of Δ*Cgrcon7* was only 1% of that of the wild type. Similar phenotypes also occurred in the *Cscon7*-deletion mutant. It has been reported that the Con7-like TF acts as a typical positive regulator in asexual sporulation in *M. oryzae* and *Verticillium* and *Fusarium* species [16,18,29,33]. From the RNA-Seq result of CgrCon7, we found that two regulatory genes of asexual development, *CgrabaA* (GLRG_00681) and *CgrwetA* (GLRG_04344), were downregulated in Δ*Cgrcon7*. Moreover, it was also observed that *CsbrlA* (CGCS363_v003651) was significantly downregulated in Δ*Cscon7*. In *Aspergillus nidulans*, three regulators, BrlA, AbaA, and WetA, constitute a central regulatory pathway of conidiogenesis, which can sequentially activate conidial production and mediate the expression of specific genes related to asexual development [34]. Therefore, we speculate that Con7 may affect conidiation by regulating the conidial developmental pathway BrlA–AbaA–WetA in *C. graminicola* and *C. siamense*. In *F. graminearum*, it has been proven that there is a direct genetic link between *FgCON7* and *FgABAA*, and the expressions of *FgABAA* and *FgWETA* were significantly decreased in Δ*Fgcon7* [18].

In addition to the decrease in conidiation, Δ*Cgrcon7* and Δ*Cscon7* also exhibited changes in conidial morphology. We speculate that deleting *con7* may influence chitin biosynthesis and alter the CWI of conidia, which further affects the shape of conidia in *C. graminicola* and *C. siamense*. Another remarkable phenotype is that disrupting *Cgrcon7* and *Cscon7* completely abolishes the ability of appressorium formation as well as hyphopodium development. In *M. oryzae*, Con7p is also required for appressorium formation, and the *con7*-deletion mutant failed to develop appressoria, probably due to the defect of chitin accumulation [15]. From the RNA-Seq result of Δ*Cgrcon7*, we noticed that a homeobox transcription factor (GLRG_00169), an ortholog of MoHox7 (Pth12) in *M. oryzae*, was downregulated in Δ*Cgrcon7* [35]. The mutant Δ*Mohox7* could not produce appressoria (hyphopodia) at the tips of germ tubes and hyphae, leading to a loss of pathogenicity [35]. CgrCon7 may be involved in regulating the expression of *GLRG_00169* and further driving the appressorium formation of *C. graminicola*.

In the pathogenicity assay, both Δ*Cgrcon7* and Δ*Cscon7* exhibited complete loss of virulence on maize and rubber tree leaves, respectively. Con7p and Con7-1 also displayed comparable roles in the pathogenicity of *M. oryzae* and *F. oxysporum* [15,17]. Given the critical role of appressoria in penetration, the defect in appressorium formation may be the main cause of the non-pathogenicity of Δ*Cgrcon7* and Δ*Cscon7*. In addition, deleting *con7* led to increased sensitivity to H_2_O_2_, suggesting that Con7 may be involved in H_2_O_2_ detoxification. The transcriptome analysis showed that some genes related to ROS detoxification, such as superoxide dismutase, peroxidase, and catalase, were significantly downregulated in Δ*Cgrcon7* and Δ*Cscon7* (Table 1). In *C. siamense*, the transcription factor gene *CgAP1* is also downregulated by CgCon7, which has been reported to play a vital role in the oxidative stress and pathogenicity of *C. gloeosporioides* (Table 1) [21]. Therefore, Con7 can also affect the pathogenicity through coordinating ROS detoxification in *C. graminicola* and *C. siamense*. From the RNA-Seq results, we also found that the expressions of several enzyme genes related to cell wall degradation were notably downregulated in Δ*Cgrcon7* and Δ*Cscon7* (Table 1). Plant pathogenic fungi usually secrete various hydrolases, such as cutinase, pectinase, and cellulose, to degrade the cell wall of hosts, contributing to their infection. Overall, our results suggest that Con7 may influence the pathogenicity of *C. graminicola* and *C. siamense* by regulating asexual sporulation, appressorium formation, ROS detoxification, and the expression of cell wall-degrading enzymes, etc.

In summary, Con7 (CgrCon7 and CsCon7) acts as a global transcription factor in *C. graminicola* and *C. siamense*, and is involved in regulating vegetative growth, CWI, oxidative stress, asexual development, appressorium and hyphopodium formation, and virulence. Future work will focus on the exploration of genes directly regulated by CgrCon7 or CsCon7, further revealing their regulatory networks and mechanisms.

## Figures and Tables

**Table 1:** Expression of some DEGs affected by CgrCon7 and CsCon7.

Functional Classification	*C. graminicola*			*C. siamense*		
	Reference Gene ID	Gene Function Annotation	Log_2_(Δ*Cgrcon7*/CgrWT)	Reference Gene ID	Gene Function Annotation	Log_2_(Δ*Cscon7*/CsWT)
ROS detoxification	GLRG_01010	Iron/manganese superoxide dismutase	−4.4	CGCS363_v014870	Catalase	−6.1
	GLRG_09508	Peroxidase	−3.6	CGCS363_v004531	Catalase-peroxidase	−5.5
	GLRG_11144	Peroxidase	−1.5	CGCS363_v006076	Catalase-1	−2.4
	GLRG_04508	Peroxidase	−1.2	CGCS363_v002325	*CgAP1*	−1.4
	GLRG_00895	Catalase	−1.6			
Chitin synthesis	GLRG_05787	Chitin synthase	1.2	CGCS363_v013013	Chitin synthase 1	2.8
	GLRG_03399	Chitin synthase	−1.2	CGCS363_v013350	Chitin synthase 3	1.7
				CGCS363_v008225	Chitin synthase 8	1.1
Cell wall degradation	GLRG_02128	Cutinase	−4.1	CGCS363_v008243	Cutinase	−1.6
	GLRG_06347	Pectin lyase	−4.4	CGCS363_v013515	Pectinesterase	−8.1
	GLRG_11038	Glucanase	−1.4	CGCS363_v014756	Pectin lyase F-1	−2.5
	GLRG_04872	β-glucosidase	−1.1	CGCS363_v002605	Endo-beta-1,4-glucanase D	−10.5
				CGCS363_v013283	Endo-1,3(4)-beta-glucanase	−4.8

## Data Availability

The original contributions presented in the study are included in the article/Supplementary Materials, further inquiries can be directed to the corresponding authors.

## Supplementary Materials

The following supporting information can be downloaded at: https://www.mdpi.com/article/10.3390/jof10070495/s1, Table S1. Primers and sequences; Figure S1. The gene-knockout strategy of Cgrcon7 (A) and Cscon7 (B); Figure S2. The expression level of con7 at different stages; Figure S3. Verification of the gene-knockout mutants and complementary strains; Figure S4. Effects of CgrCon7 and CsCon7 on conidial morphology; Figure S5. Effects of Con7 on hyphopodium formation in C. graminicola (A) and C. siamense (B); Figure S6. The qRT-PCR verification of RNA-Seq data of CgrCon7 (A) and CsCon7 (B).

## References

[1] Dean R., Van Kan J.A., Pretorius Z.A., Hammond-Kosack K.E., Di Pietro A., Spanu P.D., Rudd J.J., Dickman M., Kahmann R., Ellis J. (2012). The Top 10 fungal pathogens in molecular plant pathology. Mol. Plant Pathol..

[2] O’Connell R.J., Thon M.R., Hacquard S., Amyotte S.G., Kleemann J., Torres M.F., Damm U., Buiate E.A., Epstein L., Alkan N. (2012). Lifestyle transitions in plant pathogenic *Colletotrichum* fungi deciphered by genome and transcriptome analyses. Nat. Genet..

[3] Rogério F., Baroncelli R., Cuevas-Fernández F.B., Becerra S., Crouch J., Bettiol W., Azcárate-Peril M.A., Malapi-Wight M., Ortega V., Betran J. (2023). Population genomics provide insights into the global genetic structure of *Colletotrichum graminicola*, the causal agent of maize anthracnose. Mbio.

[4] Panaccione D.G., Vaillancourt L.J., Hanau R.M. (1989). Conidial dimorphism in *Colletotrichum graminicola*. Mycologia.

[5] Bergstrom G.C., Nicholson R.L. (1999). The biology of corn anthracnose: Knowledge to exploit for improved management. Plant Dis..

[6] Liu X., Li B., Cai J., Zheng X., Feng Y., Huang G. (2018). *Colletotrichum* species causing anthracnose of rubber trees in China. Sci. Rep..

[7] Prusky D., Lichter A. (2008). Mechanisms modulating fungal attack in post-harvest pathogen interactions and their control. Eur. J. Plant Pathol..

[8] Sukno S.A., Garcia V.M., Shaw B.D., Thon M.R. (2008). Root infection and systemic colonization of maize by *Colletotrichum graminicola*. Appl. Environ. Microbiol..

[9] Zheng W.H., Zhao X., Xie Q.R., Huang Q.P., Zhang C.K., Zhai H.C., Xu L.P., Lu G.D., Shim W.B., Wang Z.H. (2012). A conserved homeobox transcription factor Htf1 is required for phialide development and conidiogenesis in *Fusarium* species. PLoS ONE.

[10] Li X., Ke Z., Xu S., Tang W., Liu Z. (2021). The G-protein alpha subunit CgGa1 mediates growth, sporulation, penetration and pathogenicity in *Colletotrichum gloeosporioides*. Microb. Pathog..

[11] Jolma A., Yin Y., Nitta K.R., Dave K., Popov A., Taipale M., Enge M., Kivioja T., Morgunova E., Taipale J. (2015). DNA-dependent formation of transcription factor pairs alters their binding specificity. Nature.

[12] Zhu H., Situ J., Guan T., Dou Z., Kong G., Jiang Z., Xi P. (2022). A C2H2 zinc finger protein PlCZF1 is necessary for oospore development and virulence in *Peronophythora litchii*. Int. J. Mol. Sci..

[13] Shi Z., Leung H. (1995). Genetic analysis of sporulation in *Magnaporthe grisea* by chemical and insertional mutagenesis. Mol. Plant Microbe Interact..

[14] Shi Z., Christian D., Leung H. (1998). Interactions between spore morphogenetic mutations affect cell types, sporulation, and pathogenesis in *Magnaporthe grisea*. Mol. Plant Microbe Interact..

[15] Odenbach D., Breth B., Thines E., Weber R.W., Anke H., Foster A.J. (2007). The transcription factor Con7p is a central regulator of infection-related morphogenesis in the rice blast fungus *Magnaporthe grisea*. Mol. Microbiol..

[16] Tran V.T., Braus-Stromeyer S.A., Kusch H., Reusche M., Kaever A., Kuhn A., Valerius O., Landesfeind M., Asshauer K., Tech M. (2014). *Verticillium* transcription activator of adhesion Vta2 suppresses microsclerotia formation and is required for systemic infection of plant roots. New Phytol..

[17] Ruiz-Roldan C., Pareja-Jaime Y., Gonzalez-Reyes J.A., Roncero M.I. (2015). The transcription factor Con7-1 is a master regulator of morphogenesis and virulence in *Fusarium oxysporum*. Mol. Plant Microbe Interact..

[18] Shin S., Park J., Yang L., Kim H., Choi G.J., Lee Y.W., Kim J.E., Son H. (2024). Con7 is a key transcription regulator for conidiogenesis in the plant pathogenic fungus *Fusarium graminearum*. Msphere.

[19] Li X., Liu S., Zhang N., Liu Z. (2021). Function and transcriptome analysis of an oligopeptide transporter CgOPT2 in the rubber anthracnose fungus *Colletotrichum gloeosporioides*. Physiol. Mol. Plant Pathol..

[20] Murray M.G., Thompson W.F. (1980). Rapid isolation of high molecular weight plant DNA. Nucleic Acids Res..

[21] Li X., Wu Y., Liu Z., Zhang C. (2017). The function and transcriptome analysis of a bZIP transcription factor CgAP1 in *Colletotrichum gloeosporioides*. Microbiol. Res..

[22] Gao J., Zhou S., Tang W., Wang J., Liu H., Zhang Y., Wang L., Li X., Liu Z. (2022). The velvet proteins CsVosA and CsVelB coordinate growth, cell wall integrity, sporulation, conidial viability and pathogenicity in the rubber anthracnose fungus *Colletotrichum siamense*. Microbiol. Res..

[23] Werner S., Sugui J.A., Steinberg G., Deising H.B. (2007). A chitin synthase with a myosin-like motor domain is essential for hyphal growth, appressorium differentiation, and pathogenicity of the maize anthracnose fungus *Colletotrichum graminicola*. Mol. Plant Microbe Interact..

[24] Shelp B.J., Bown A.W., McLean M.D. (1999). Metabolism and functions of gamma-aminobutyric acid. Trends Plant Sci..

[25] Livak K.J., Schmittgen T.D. (2001). Analysis of relative gene expression data using real-time quantitative PCR and the 2^−ΔΔCT^ method. Methods.

[26] Rogers S., Wells R., Rechsteiner M. (1986). Amino acid sequences common to rapidly degraded proteins: The PEST hypothesis. Science.

[27] Zou S., Wang H., Li Y., Kong Z., Tang D. (2018). The NB-LRR gene *Pm60* confers powdery mildew resistance in wheat. New Phytol..

[28] Wolfe S.A., Nekludova L., Pabo C.O. (2000). DNA recognition by Cys2His2 zinc finger proteins. Annu. Rev. Biophys. Biomol. Struct..

[29] Chen S., Li P., Abubakar Y.S., Lu P., Li Y., Mao X., Zhang C., Zheng W., Wang Z., Lu G.D. (2024). A feedback regulation of FgHtf1-FgCon7 loop in conidiogenesis and development of *Fusarium graminearum*. Int. J. Biol. Macromol..

[30] Pareja-Jaime Y., Martin-Urdiroz M., Roncero M.I.G., Gonzalez-Reyes J.A., Roldan M.D.R. (2010). Chitin synthase-deficient mutant of *Fusarium oxysporum* elicits tomato plant defence response and protects against wild-type infection. Mol. Plant Pathol..

[31] Silva A.D., Aliyeva-Schnorr L., Wirsel S.G.R., Deising H.B. (2022). Fungal pathogenesis-related cell wall biogenesis, with emphasis on the maize anthracnose fungus *Colletotrichum graminicola*. Plants.

[32] Odenbach D., Thines E., Anke H., Foster A.J. (2009). The *Magnaporthe grisea* class VII chitin synthase is required for normal appressorial development and function. Mol. Plant Pathol..

[33] Cao H.J., Huang P.Y., Zhang L.L., Shi Y.K., Sun D.D., Yan Y.X., Liu X.H., Dong B., Chen G.Q., Snyder J.H. (2016). Characterization of 47 Cys(2)-His(2) zinc finger proteins required for the development and pathogenicity of the rice blast fungus *Magnaporthe oryzae*. New Phytol..

[34] Park H.S., Yu J.H. (2012). Genetic control of asexual sporulation in filamentous fungi. Curr. Opin. Microbiol..

[35] Kim S., Park S.Y., Kim K.S., Rho H.S., Chi M.H., Choi J., Park J., Kong S., Park J., Goh J. (2009). Homeobox transcription factors are required for conidiation and appressorium development in the rice blast fungus *Magnaporthe oryzae*. PLoS Genet..

