# Supplementary material for: The C2H2 Transcription Factor Con7 Regulates Vegetative Growth, Cell Wall Integrity, Oxidative Stress, Asexual Sporulation, Appressorium and Hyphopodium Formation, and Pathogenicity in Colletotrichum graminicola and Colletotrichum siamense
Source: J Fungi (Basel). 2024 Jul 17;10(7):495. doi: 10.3390/jof10070495 (PMC11277718; doi:10.3390/jof10070495)
Supplement: Supplementary file 1 [file jof-10-00495-s001.zip › jof-3081908-supplementary.pdf]

Table S1. Primers and sequences.

Primer	Primer sequence（5'-3'）	Use in this study
Cgrcon7UF	GGGGTACCAAGCATCAGCGCCCACCATCCAC	5' flanking sequence of *Cgrcon7*
Cgrcon7UR	GCTCTAGACAAGCCAACGATGGGTTCGCAGG	
Cgrcon7F	TCAGGTCCCGATGCCCAAGTCT	Validation of *ΔCgrcon7*
Cgrcon7R	TCCTCGGCCTTTCGCTGTGCTT	
Cgrcon7DF	CGGAATTCTCTAGCCACTGAAGCAGGCTGAC	3' flanking sequence of *Cgrcon7*
Cgrcon7DR	GGGGTACCTTGACACGGGCTGAAGGGAACTC	
Cgrcon7UU	TCGACTTCGGCACACAGAGAAATCC	Validation of *ΔCgrcon7*
pUC18-PI	GTCCTCGTTCCTGTCTGCTAATAAG	
pUC18-PI1	GGCACCCCAGGCTTTACACTTTATG	Validation of *ΔCgrcon7*
Cgrcon7DD	TTGGCTACCCAACTCAACCTTTGCT	
Cgrcon7hbF	CGGAATTCCCTCGGCTACACCTCACATCAA	Complementary sequence of *Cgrcon7*
Cgrcon7hbR	GGGGTACCAATCCTGTGCCCTCGCCTTCTA	
Cgrcon7OF	ATGTTCGTGGTCCCAACGCA	ORF of *Cgrcon7*
Cgrcon7OR	TCAGCATGCTCCAGGAGTCT	
Cgrcon7-GFPF	GGGGTACCATGTTCGTGGTCCCAACGCA	Construction of *Cgrcon7-GFP* fusion
Cgrcon7-GFPR	GCTCTAGAGCATGCTCCAGGAGTCTTCA	vector
Cgrcon7-qF	AACAAGCAAGCAGTCCGTCT	qRT-PCR for *Cgrcon7*
Cgrcon7-qF	CATAGGCAGGGTGCTGGTAG	
Tub-qF	GCCAGTGCGGTAACCAGATTG	qRT-PCR for tubulin gene of *C.*
Tub-qR	GAAGTTGTCGGGGCGGAAGAG	*graminicola* and *C. siamense*
Cscon7UF	CGGGATCCACACGCACAAGCTCCCGTCAATC	5' flanking sequence of *Cscon7*
Cscon7UR	AACTGCAGACGTCTTGGTCGCAAAGTATGGC	
Cscon7F	AGTTCGCTTCTGTCGTCCAT	Validation of *ΔCscon7*
Cscon7R	CGTAGGGTGACTGAGGTTGG	
Cscon7DF	GCTCTAGAAGCACAGGAGGCTAACTGCACAG	3' flanking sequence of *Cscon7*
Cscon7DR	CGGGATCCATCTCGCCACTCCAAAGGCACAT	
Cscon7UU	CAAGTTCACACCCACGCTACGCTAC	Validation of *ΔCscon7*
PI	CAGGGTTTTCCCAGTCACGACGTTG	
PI1	GTATGTTGTGTGGAATTGTGAGCGG	Validation of *ΔCscon7*
Cscon7DD	AAGTTGTAGAGCTTAGTGAACCGGG	
Cscon7hbF	CGGAATTCCACGCTGCTCCCAAGTTCACACC	Complementary sequence of *Cscon7*
Cscon7hbR	GCTCTAGAGCCAACCCTGTGCTTTCGCCTTC	
Cscon7OF	ATGTTCTTGGTTCCAACGCA	ORF of *Cscon7*
Cscon7OR	CTAACGTGAGCTGTACATGC	
Cscon7-GFPF	CGGAATTCATGTTCTTGGTTCCAACGCAA	Construction of *Cscon7*-GFP fusion
Cscon7-GFPR	GCGTCGACACGTGAGCTGTACATGCTCT	vector

Cscon7-qF	GCACTCATACCAGCACCCTT	qRT-PCR for *Cscon7*
Cscon7-qF	ACCTGGTTAGGTCGTCCCTG	

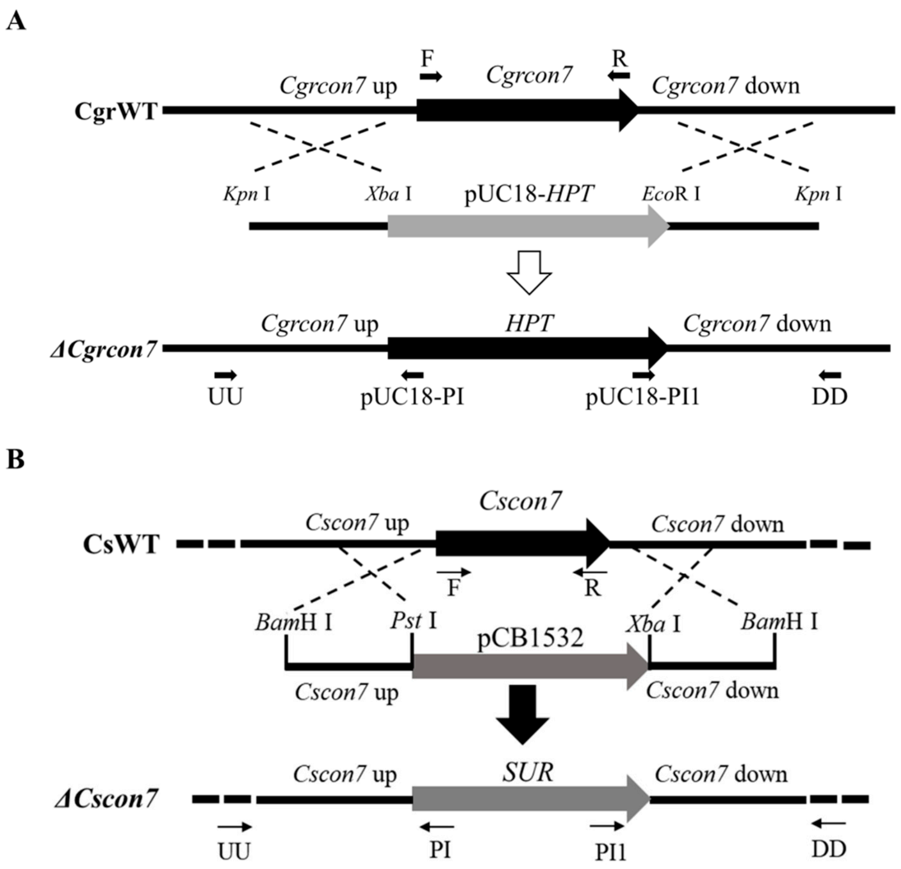

Figure S1. The gene-knockout strategy of *Cgrcon7* (A) and *Cscon7* (B). *HPT* is hygromycin B resistance gene. *SUR* is the chlorimuron-ethyl resistance gene. The small black arrows indicate the primers used to verify gene-knockout mutants.

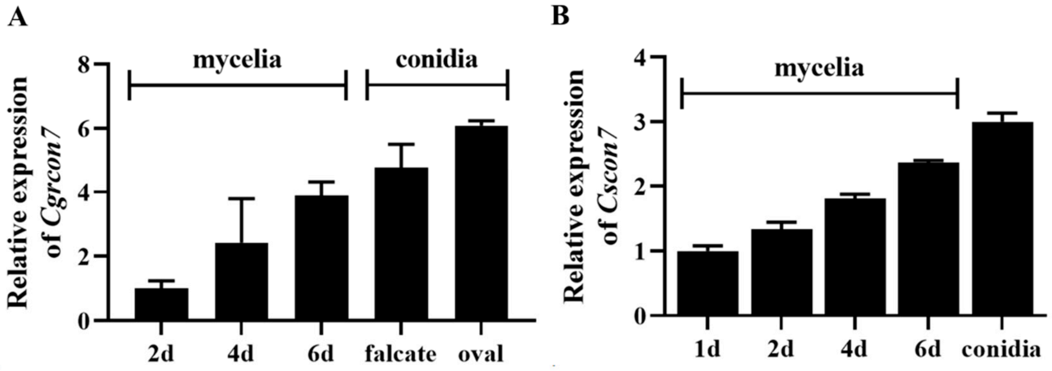

Figure S2. The expression level of *con7* at different stages. (A) Relative expression of *Cgrcon7* in *C. graminicola*. (B) Relative expression of *Cscon7* in *C. siamense*.

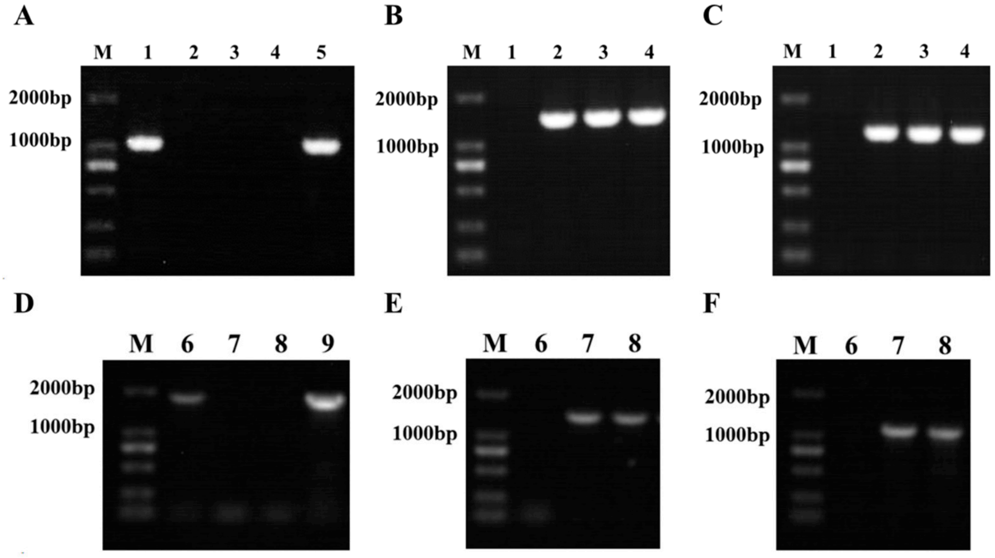

Figure S3. Verification of the gene-knockout mutants and complementary strains. A: PCR results of Cgrcon7F / Cgrcon7R. B: PCR results of Cgrcon7UU / pUC18-PI. C: PCR results of Cgrcon7DD / pUC18-PI1. D: PCR results of Cscon7F / Cscon7R. E: PCR results of Cscon7UU / PI; F: PCR results of Cscon7DD / PI1. M: DL2000 DNA marker, 1: CgrWT, 2: *ΔCgrcon7*-10, 3: *ΔCgrcon7*-13, 4: *ΔCgrcon7*-17, 5: *ΔCgrcon7*-C, 6: CsWT, 7: *ΔCscon7*-12, 8: *ΔCscon7*-15, 9: *ΔCscon7*-C.

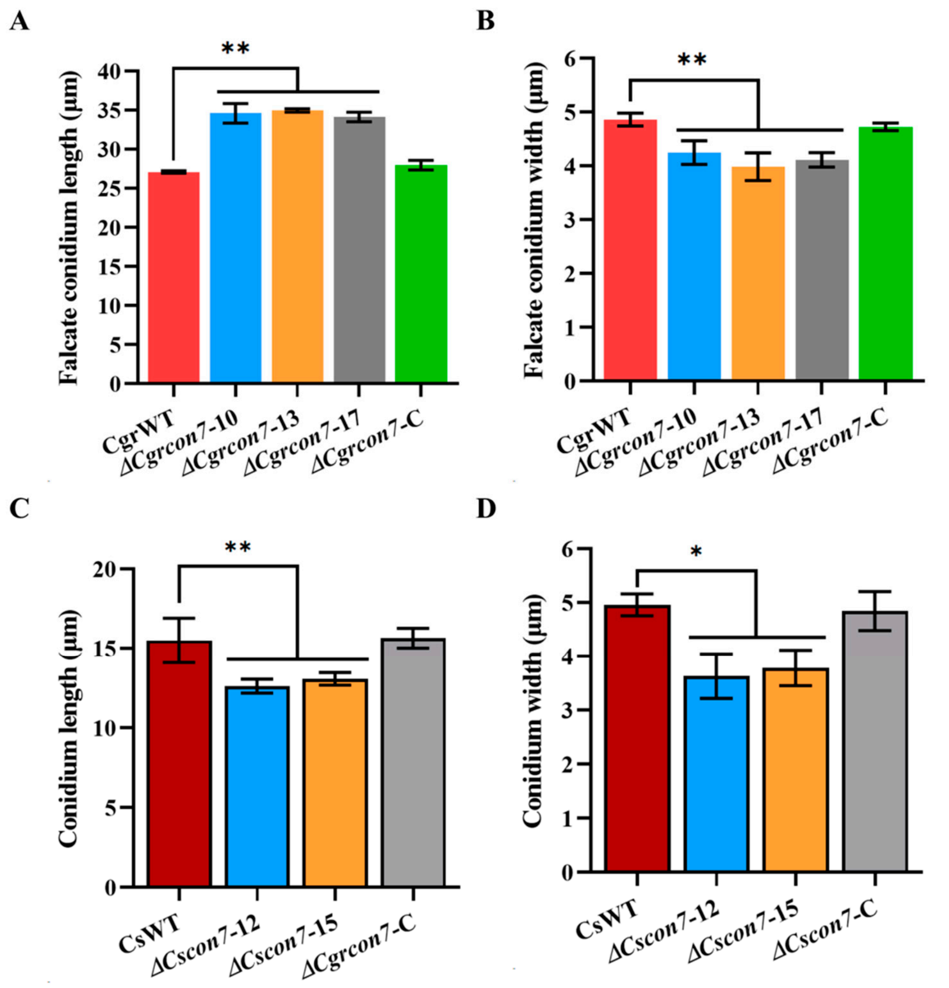

Figure S4. Effects of CgrCon7 and CsCon7 on conidial morphology. Statistical analyses of falcate conidium length (A) and width (B) of *C. graminicola* strains. Statistical analyses of conidium length (C) and width (D) in *C. siamense* strains. *: significant level at $P < 0.05$; **: significant level at $P < 0.01$.

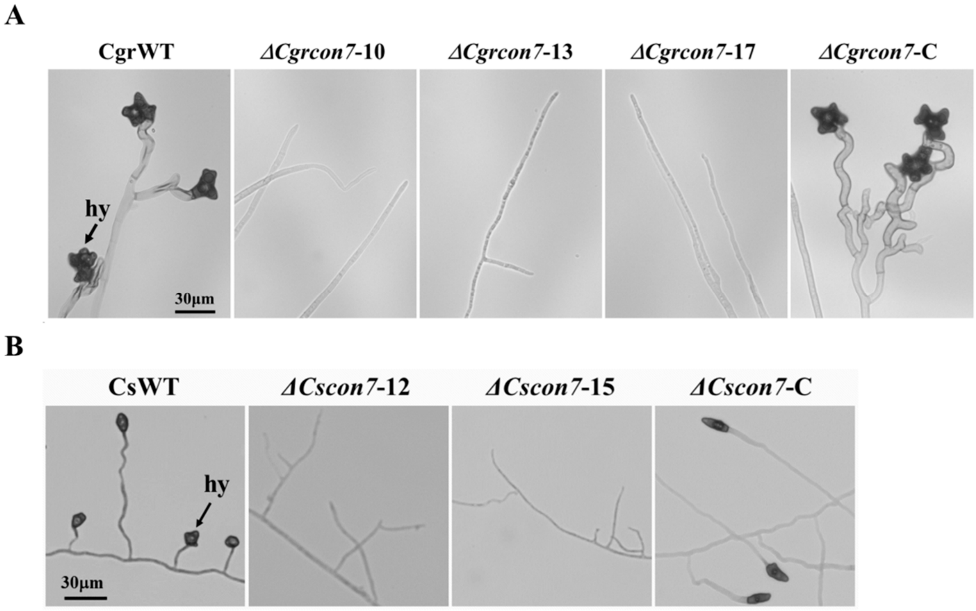

Figure S5. Effects of Con7 on hyphopodium formation in *C. graminicola* (A) and *C. siamense* (B). hy: hyphopodium.

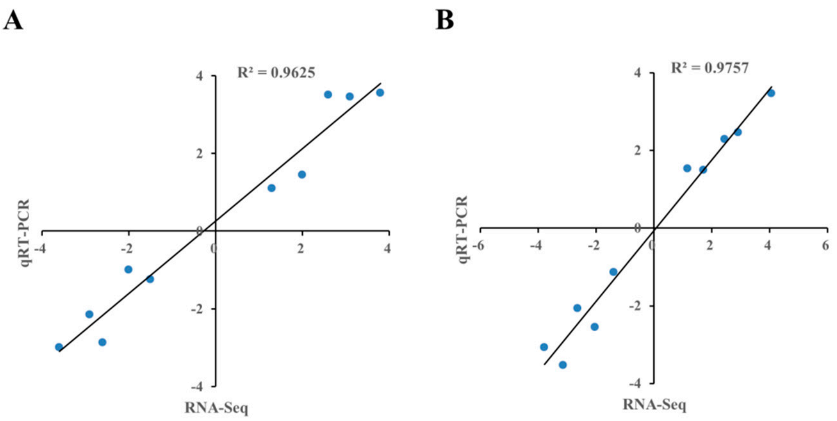

Figure S6. The qRT-PCR verification of RNA-Seq data of CgrCon7 (A) and CsCon7 (B).
